# RETRACTED: Jeong et al. Protective Effects of Fermented Oyster Extract Against RANKL-Induced Osteoclastogenesis Through Scavenging ROS Generation in RAW 264.7 Cells. *Int. J. Mol. Sci.* 2019, *20*, 1439

**DOI:** 10.3390/ijms27135675

**Published:** 2026-06-24

**Authors:** Jin-Woo Jeong, Sung Hyun Choi, Min Ho Han, Gi-Young Kim, Cheol Park, Su Hyun Hong, Bae-Jin Lee, Eui Kyun Park, Sung Ok Kim, Sun-Hee Leem, You-Jin Jeon, Yung Hyun Choi

**Affiliations:** 1 Freshwater Bioresources Utilization Bureau, Nakdonggang National Institute of Biological Resources, Sangju 37242, Republic of Korea; jwjeong@nnibr.re.kr; 2 Department of System Management, Korea Lift College, Geochang 50141, Republic of Korea; hyunle6869@hanmail.net; 3 National Marine Biodiversity Institute of Korea, Seocheon 33662, Republic of Korea; mhhan@mabik.re.kr; 4 Department of Marine Life Sciences, School of Marine Biomedical Sciences, Jeju National University, Jeju 63243, Republic of Korea; immunkim@jejunu.ac.kr (G.-Y.K.); youjinj@jejunu.ac.kr (Y.-J.J.); 5 Department of Molecular Biology, College of Natural Sciences, Dong-eui University, Busan 47340, Republic of Korea; parkch@deu.ac.kr; 6 Department of Biochemistry, Dong-eui University College of Korean Medicine, Busan 47227, Republic of Korea; hongsh@deu.ac.kr; 7 Anti-Aging Research Center, Dong-eui University, Busan 47227, Republic of Korea; 8 Marine Bioprocess Co., Ltd., Busan 46048, Republic of Korea; hansola82@hanmail.net; 9 Department of Oral Pathology and Regenerative Medicine, School of Dentistry, Kyungpook National University, Daegu 41940, Republic of Korea; epark@knu.ac.kr; 10 Department of Food Science and Biotechnology, College of Engineering, Kyungsung University, Busan 48434, Republic of Korea; theresa10000@naver.com; 11 Department of Biological Sciences, College of Natural Science, Dong-A University, Busan 49315, Republic of Korea; shleem@dau.ac.kr

The journal retracts the article titled “Protective Effects of Fermented Oyster Extract against RANKL-Induced Osteoclastogenesis through Scavenging ROS Generation in RAW 264.7 Cells” [1], cited above.

Following publication, concerns were brought to the attention of the Editorial Office regarding the integrity of several figures published in this article [1].

In accordance with standard journal procedures, the Editorial Office and Editorial Board conducted an investigation that confirmed inappropriate editing in several images and duplication with previously published figures in [2], produced by a similar authorship group and depicting different experimental conditions. Although the authors cooperated during the investigation, the explanations and supporting materials provided were not deemed sufficient by the Editorial Board to resolve the identified integrity issues and establish the provenance of the images.

Consequently, the Editorial Board has lost confidence in the reliability of the findings and decided to retract this publication [1], as per MDPI’s retraction policy.

This retraction was approved by the Editor-in-Chief of the journal *IJMS*.

The authors have been informed of this retraction and have indicated their disagreement with this decision.
